# Long-term therapeutic outcome of ophthalmic complications following endoscopic sinus surgery

**DOI:** 10.1097/MD.0000000000004896

**Published:** 2016-09-23

**Authors:** Heping Wu, Tao Shen, Jingchang Chen, Jianhua Yan

**Affiliations:** The State Key Laboratory of Ophthalmology, Zhongshan Ophthalmic Center, Sun Yat-sen University, Guangzhou, The People's Republic of China.

**Keywords:** endoscopic sinus surgery, ocular motility, orbital hemorrhage, paranasal sinuses, strabismus, visual loss

## Abstract

Ophthalmic complications associated with endoscopic sinus surgery (ESS) are quite rare. There is a paucity of reliable data and limited experience on the clinical findings and treatments of these injuries. Our study here is to characterize the types of orbital injury following ESS, in particular extraocular muscle injury, and to evaluate the long-term therapeutic outcomes as compiled from a relatively large sample of Chinese patients.

A series of 27 patients (21 males and 6 females; mean age = 42.6 years, ranges: 10–60 years) were retrospectively reviewed. The mean duration of orbital complication was 6.6 months (ranges: 1 day to 24 months). The right eye was affected in 19 patients and the left in 8 patients. All patients had various extraocular muscle dysfunction, including contusion, oculomotor nerve damage, muscle entrapment, muscle transection, and muscle destruction. All patients subjected to strabismus surgery showed an obvious reduction in deviation. Three patients achieved orthophoria without any surgery during the period of observation. All patients displayed mild to complicated orbital hemorrhage that often disappeared within 2 weeks. Optic nerve injury occurred in 29.6% of patients and vision damage in these patients was often irreversible.

All patients with ophthalmic complications after ESS had strabismus and extraocular muscle dysfunction. Timing and type of strabismus surgery performed depended on the severity and number of muscles involved as well as the type of injury. This surgery is less effective in cases of restriction factor adhesion and/or entrapment as compared to that of patients with other types of strabismus. Orbital hemorrhages were usually resolved spontaneously, but optic nerve injury was mostly irreversible.

## 1 Introduction

Currently, endoscopic sinus surgery (ESS) has become the surgical technique of choice for the treatment of medically resistant infectious sinusitis in China.^[1,2]^ Recent technical improvements in ESS have enhanced the safety of this procedure; however, the risk of ophthalmic complications is still possible as a result of the narrow surgical field and the intimate anatomic relationship between the sinuses and orbit. Orbital complications of ESS include medial wall defect, orbital hemorrhage, optic nerve damage, nasolacrimal duct injury, and extraocular muscle injury.^[3–6]^ The management of these patients can be challenging due to the low incidence and complexity of injuries. Although there have been some studies describing the clinical features and management of orbital complications in ESS, most are short anecdotal reports.^[5–10]^ There is a paucity of reliable data and limited experience on the clinical findings and treatments of orbital injuries after ESS. The purpose of this study is to characterize the types of orbital injury following ESS, in particular extraocular muscle injury, and to evaluate the long-term therapeutic outcomes as compiled from a relatively large sample of 27 Chinese patients.

## 2 Materials and methods

A retrospective review was performed on 27 cases of Chinese patients who presented with ophthalmic complications after ESS that were treated at the Zhongshan Ophthalmic Center of Sun Yat-sen University in Guangzhou, China between January 1, 2003 and August 31, 2014. The ethics committee of the Zhongshan Ophthalmic Center approved this retrospective study, and this study was conducted according to the principles expressed in the Declaration of Helsinki. The committee specifically waived the need for consent. Each patient underwent complete ophthalmic examinations, including the best corrected visual acuity, intraocular pressure, anterior segments, fundi, ocular motility, diplopia test, and orbital imaging. Computed tomography (CT) and/or magnetic resonance imaging (MRI) of the involved orbits were available for each patient.

In patients with medial wall defect or fracture, surgical repair of orbital fracture is technically challenging. With a small medial wall defect or fracture, the medial rectus muscle or orbital tissues are not entrapped and therefore no need for surgical repair of the bone defect. If a large portion of bone damage exists or an entrapment or transection of the muscle is present, we repair the bony defect with an implant and release the scarring tissue or reattachment the medial rectus with a silk suture through an anterior orbitotomy.

In patients with muscle damage, deviations for near and distance were determined using both a major synoptophore and an alternate prism cover test in 9 gaze positions. The range of adduction deficits were recorded at the version test using the following scales: 0 indicated full adduction – free arrival of medial limbus at the medial canthal area, −4 indicates that the medial limbus could not past midline, and −1, −2, and −3 indicated that the eye could rotate nasally from the midline to 75%, 50%, and 25% of full adduction, respectively.^[11]^ A forced duction test and a forced generation test using toothed forceps were performed to determine restriction or paresis of the affected muscle. If the globe could not be passively rotated further than the patient's effort, restriction was diagnosed; if passive rotation was possible, paresis was diagnosed.^[3]^

Surgical correction for strabismus after ESS is difficult and quite complicated. It should be individualized for each case, based on the type of muscle injury, the time at presentation, and other orbital complications. In patients with a muscle transection, reattachment of that muscle was possible based on image findings, and involved reattaching the ends of the affected muscle through orbital exploration. Usually, partial-tendon vertical rectus muscle transposition toward the insertion site of the medial rectus combined with recession of lateral rectus was performed for medial rectus injury. A single posterior fixation suture was placed at the sclera at 8 mm posterior to each upper and lower corner of medial rectus insertion. When the time interval between the presentation and sinus surgery was greater than 6 months, lateral rectus recession and a Jensen procedure could be performed. When the involved muscle was capable of moving the eye across the midline, then rectus muscle recession and resection was performed. If the deviation was small, then only resection of the affected muscle or recession of the antagonistic muscle may be required. In severe cases, a nasal periosteal globe fixation procedure was performed for medial rectus injuries.

In patients with optic nerve damage, optic neuropathy could result from direct trauma of resulting from the surgery, damage to local blood supplies, or optic nerve compression by a retrobulbar hematoma. In such circumstances, an examination of the best corrected visual acuity, visual field, and orbital imaging (CT or MRI) must be performed. If an optic nerve transection is present, there is no specific treatment. For other conditions, the use of high dose corticosteroids should be administered within the 1st 8 hours of optic nerve injury (e.g., 1000 mg methylprednisolone sodium succinate per day for 3 days). Optic canal decompression through an intranasal endoscopic approach in combination with high-dose corticosteroids was used for patients with rapid re-reduction of vision after nose surgery.

In patients with orbital hemorrhage or hematoma, most presented with subconjunctival bleeding, mild periorbital ecchymoses, and periorbital bruising due to small artery damage and fracture to the lamina papyracea. In such circumstances, conservative observation and avoidance of nose blowing is recommended. However, in the few patients with severe orbital hemorrhage, urgent management is required to avoid permanent visual loss. Symptoms and signs include extreme pain, remarkable proptosis, severe chemosis, and periorbital ecchymosis. Ocular motility is remarkably restricted. Visual field and vision loss can occur and there is a risk of blindness. Initial management involves removal of nasal packing and control of any epistaxis. When normal vision has been restored and the orbital hematoma is stable, systemic corticosteroids and mannitol can help to reduce intraorbital pressure, proptosis, and paraorbital inflammation. If this conservative treatment fails, orbital decompression and/or endoscopic optic nerve decompression should be considered.

## 3 Results

Twenty-seven patients were identified with orbital injury resulting from ESS. All patients were referred with eye pain, eye deviation, diplopia, proptosis, decreased vision, and orbital hemorrhage after ESS. The mean duration of orbital complication was 6.6 months (ranges: 1 day to 24 months) at the time of presentation. Twenty-one patients were male and 6 female, with a mean age of 42.6 years (ranges: 1060 years). The right eye was affected in 19 patients (70.4%) and the left eye in 8 (29.6%) patients. All involved eyes had no record of previous eye disease history. Follow-up time from the management of ophthalmic complications averaged 24.3 months (ranges: 6–119 months).

All referred patients had strabismus and extraocular muscle damage. A variety of insults to the extraocular muscles were present, ranging from partial to complete transection, complete destruction, entrapment in scar tissue, contusion, and injury to the oculomotor nerves. Table 1 provides a summary of the extraocular muscles involved, the preoperative alignment, the corresponding injury/surgery, and postoperative alignment of the patients. Injury to only 1 extraocular muscle was present in 21 of 27 patients (77.8%), while 4 (14.8%) had injury to 2 muscles, 1 (3.7%) had involvement of 3 muscles, and 1 (3.7%) had involvement of 4 muscles. The medial rectus was the most commonly involved muscle (25 of 27 cases, 92.6%), followed by the inferior rectus (6 of 27 cases, 22.2%), superior and inferior obliques (2 of 27 cases, 7.4%), and superior rectus and levator palpebrae superioris (1 of 27cases, 3.7%). On ocular motility examination, all patients with medial rectus injury show a complete absence of adduction, and the eye could not move beyond the midline preoperatively. The angle of exotropia (XT) ranged from 15 prism diopters (PDs) to 140 PD. Patients with inferior rectus injury showed an angle of hypertropia from 10 to 20 PD. Two patients developed esotropia of 4 and 30 PD, respectively.

**Table 1:**
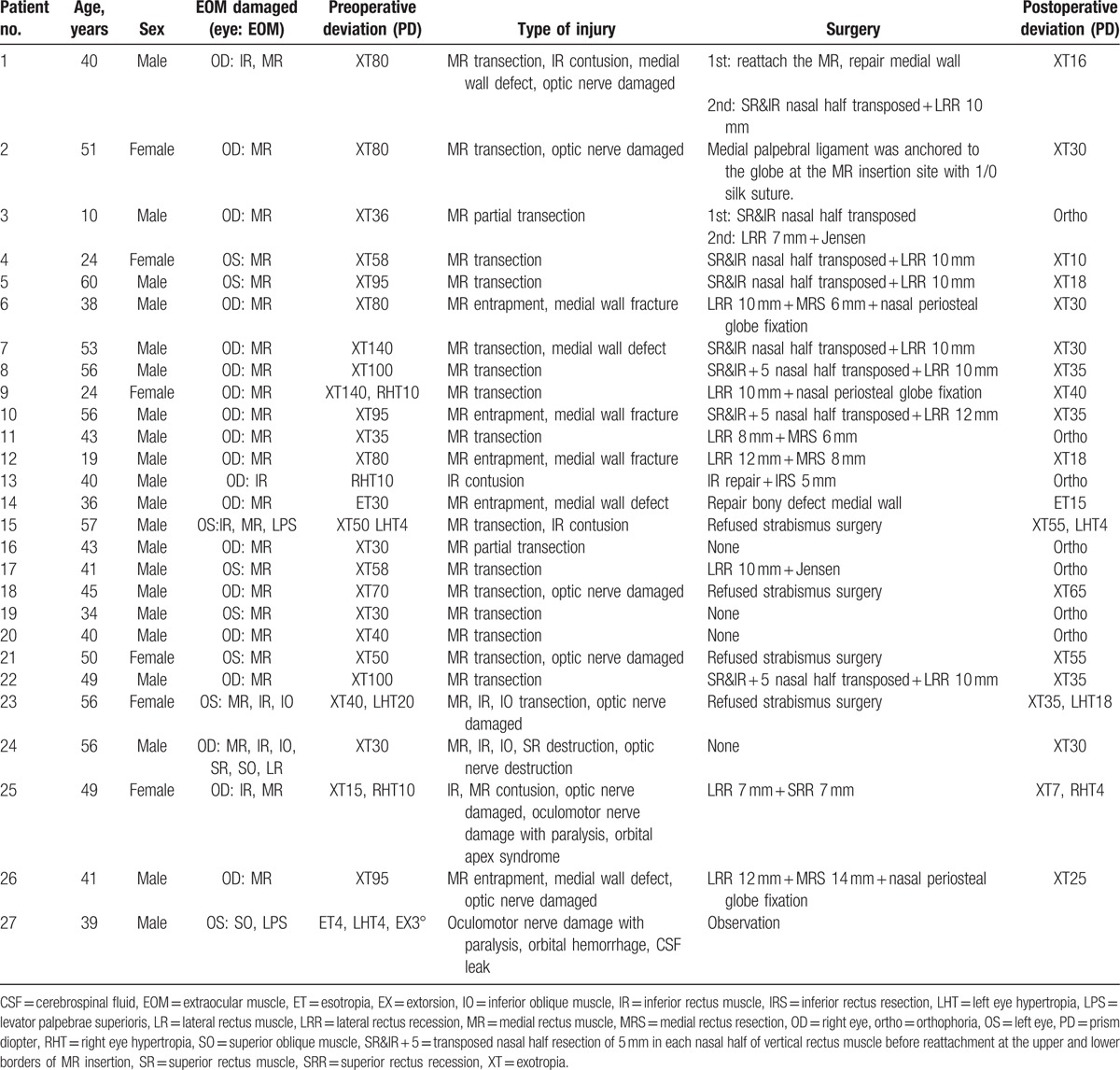
The clinical features and therapeutic outcomes for orbital injury associated with endoscopic sinus surgery.

The forced duction test was performed on all patients. All affected recti muscles demonstrated moderate (15/27, 55.6%) or remarkable (12/27, 44.4%) restriction in the direction of suspected limited rotation. The 12 patients who showed severe limitations (could not cross the midline) require special attention as the strabismus surgery in these patients failed to achieve a good alignment in the primary position due to the severe nature of the restriction.

A transconjunctival medial orbitotomy was performed under general anesthesia for repairing orbital wall defects in 2 cases (case 1 and case 14). In case 1, the medial wall defect was repaired, the medial rectus recovered and was reattached to the globe. XT decreased from 80 to 30 PD. Six months later, the angle of XT increased to 45 PD. Subsequently, the XT was treated with superior and inferior recti nasal half transpositions and lateral rectus recession (10 mm). The final postoperative deviation was 16 PD XT. In case 14, orbital fat was adherent to the medial wall defect with incarceration of the medial rectus. The tissues were freed from the defect and the medial wall repaired with a hydroxylapatite sheet. Immediately after surgery the deviation of esotropia 30 PD was reduced to esotropia 15 PD, enabling the patient the capacity to fuse in the primary position by a slight head turn.

**Figure 1:**
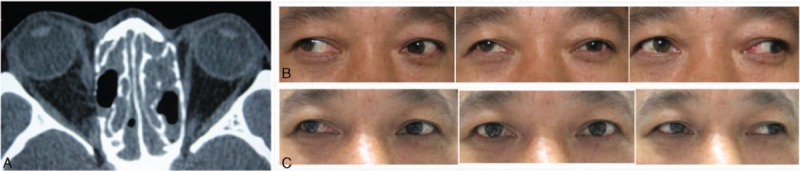
Clinical photos of case 11. (A) Axial computed tomography view showing right medial rectus transection in the posterior 3rd with small amounts of the posterior muscle segment remaining intact. (B) Preoperative clinical photos showing right exotropia of 35 prism diopters and obvious limitations of adduction. (C) Postoperative clinical photos after lateral rectus recession 8 mm and medial rectus resection 6 mm. No horizontal heterotropia was noted postoperatively in the primary position with only mild limitations of adduction.

Sixteen of the 27 patients (59.3%) received 1 strabismus surgery and 1 patient (1/27, 3.7%) required 2 strabismus surgeries for deviation correction. Surgical intervention for strabismus was not performed in the remaining 10 patients (37.0%). Superior and inferior recti nasal half transpositions alone or combined with lateral rectus recession were performed in the 8 cases with medial rectus injury. Three of these 8 cases underwent a 5 mm resection of both superior and inferior recti before reattachment at the upper and lower borders of the medial rectus, while another 3 cases required an augmented fixation suture with the nasal half of the split muscle reattached at the upper and lower borders of the medial rectus 8 mm posteriorly. In these 8 cases, the average angle of XT was 88 PD (ranges: 36–140 PD) preoperatively. Following surgery, the XT decreased to 22.4 PD (ranges: 0–35 PD). In case 3, only a superior and inferior recti nasal half transposition was performed initially, and XT decreased from 36 to 10 PD. Seven months later, the angle of XT returned to 20 PD. Therefore, a 2nd strabismus surgery was performed using the Jensen procedure and a 7 mm lateral rectus recession. Postoperative alignment achieved orthophoria. Interestingly, in case 17 with a medial rectus injury, the use of the Jensen procedure combined with lateral rectus recession resulted in an excellent surgical outcome (from a preoperative deviation of XT 58PD to postoperative orthophoria), provided that the time interval between ESS and strabismus surgery was greater than 6 months. Eight patients had strabismus surgery of rectus recession and resection alone, or combined with periosteal globe fixation. In these 8 cases, the average angle of deviation was 66.9 PD (ranges: 10–140 PD) preoperatively. Following surgery, the mean deviation decreased to 18.8 PD (ranges: 0–40 PD), ocular alignment improved in all patients and 2 achieved orthophoria (Fig. 1). Ten of the 27 patients (37.0%) in this study were not subjected to strabismus surgery. To our surprise, 3 of 10 cases with XT of 30, 30, and 40 PD achieved orthophoria upon final follow-up examination.

Optic nerve injury occurred in 8 patients (29.6%), and the impairing vision ranged from complete blindness to 6/10. Two patients with severe optic nerve destruction showed no light perception. Two patients had a vision of only hand move/30 cm. The remaining 4 patients had visions of 2/100, 1/10, 2/10, and 6/10. The vision damage was irreversible for all but one of these patients whose vision recovered from 1/10 to 20/20 after emergency decompression of the orbit and optic nerve.

Patients with mild or complicated orbital hemorrhage or hematoma showed subconjunctival bleeding, mild periorbital ecchymoses, and periorbital bruising. These symptoms often disappeared within 2 weeks. However, severe orbital hemorrhage occurred in 1 patient and visual acuity decreased from 20/20 to 1/10. After an emergency decompression surgery of the orbit and optic nerve, normal vision was restored. In 1 patient (27), a small cerebrospinal fluid leak and levator palpebrae superioris paralysis cured spontaneously after conservative treatment.

## 4 Discussion

ESS typically represents a safe surgical technique of choice for sinus diseases with a major complication rate of 1% to 2% as defined by orbital hemorrhage, blindness, diplopia, hemorrhage, and skull base defects.^[12]^ Usually, a small orbital wall defect, limited orbital hemorrhage, and various degrees of damage to extraocular muscles comprise the most common ophthalmic complications, which may then leave the patient with strabismus and double vision (diplopia).

Due to the close contact with the lamina papyracea, the medial rectus was the most commonly traumatized muscle in our review, which is, similar to that observed in previous reports.^[9–13]^ In addition, the inferior rectus, inferior oblique, superior oblique, and levator muscles may also be damaged in some of these cases. There were 2 patients with damage to all 4 recti and 2 oblique muscles which were the result of a nearly complete destruction of the posterior orbital structures in 1 case and severe orbital hemorrhage with orbital apex syndrome in the other case. To our knowledge, there are no previous reports of levator, superior rectus, and lateral rectus muscle damage as a complication of ESS. These injured extraocular muscles may have a contusion, partial or complete transection, destruction, neurovascular interruption, extraocular muscle, and orbital soft-tissue entrapment or development of adhesion with adjacent structures.^[7–10]^ However, disruption of eye movement can also be caused by irregular scars resulting from destruction of the orbital fascia, in the absence of a direct muscular injury.^[12]^ Huang et al^[6]^ published the largest series of ESS-related medial rectus muscle injures. In this report, 4 common patterns of medial rectus injury were identified from their sample of 30 patients: I (complete transection), II (partial transection), III (intact/mildly contusion with entrapment), and IV (intact/mildly contusion without entrapment).

To achieve a thorough assessment of the affected orbit, high-resolution CT evaluation and/or MRI of the orbit should be performed in 3 planes.^[5,6,12,14–16]^ Orbital imaging is essential for determining the extent and nature of the orbital injury, including extraocular muscles, optic nerve, orbital wall defect, and hemorrhage. The added benefit of multipositional MRI is its capacity to demonstrate muscle contractility, which can then enable a more precise diagnosis in complicated cases and help to devise the most suitable treatment plan.^[3]^ However, it should be noted that motility limitations can be distinctly higher than that revealed by the damage observed by imaging, due to adhesion between the periorbital, fat, sclera, and extraocular muscles.^[5]^

Management of orbital complications resulting from ESS is challenging and difficult. Medial wall defects are often small and posterior and therefore require no need for repair, especially when no muscle entrapment or adhesion exist. Retrieval of a transected muscle is difficult as the posterior muscle portion may retract posteriorly and the muscle can be entrapped in scar tissue or form adhesion with adjacent structures.^[3]^ Successful reconstruction of the medial rectus can be challenging.^[4]^ However, if the medial wall defect is large, anterior, or involves muscle entrapment, it should be repaired whenever possible. As suggested by Thacker et al,^[3]^ in cases of transection injury, the medial rectus can be reattached through an anterior orbitotomy if the remaining posterior stump is longer than 20 mm. An orbital surgical approach is often needed to reattach the transected muscle. Repair of the medial orbital wall usually cannot prevent secondary scar formation.^[11,17,18]^ In our experience, recovery of the medial rectus was abandoned in 2 cases where severe entrapment and/or adhesion were present and strabismus surgery was employed in these cases. Two of the 27 cases in our study had their medial wall defect repaired through a transconjunctival orbitotomy. In case 1, the transected medial rectus recovered and was reattached to the globe. In case 14, incarceration of the medial rectus was freed from the defect and the medial wall repaired. Muscle recovery is not always possible if the damage is severe or the muscle becomes entrapped in scar tissue.^[3]^

Timing and type of strabismus surgery depends on the severity and number of muscles involved as well as the type of injury. This surgery is less effective in cases of restriction factor adhesion and/or entrapment as compared to that of patients with other types of strabismus. Several operations may be necessary in two thirds of these cases as reported by others.^[3,17]^ In cases with damaged medial rectus muscles, partial-tendon vertical rectus muscle transposition combined with recession of lateral rectus is the surgery of choice. The Jensen procedure combined with lateral rectus recession can be used if the interval between injury and presentation is greater than 6 months. Recession of the lateral rectus combined with medial rectus resection also produced favorable results in some patients. In cases with severe muscle adhesion or with coexistent injuries involving the inferior and superior recti muscles, it will be necessary to perform a nasal periosteal globe fixation procedure to achieve primary alignment, when recession and/or resection of rectus was not efficacious. Although rare, anterior segment ischemia is a potentially serious complication of strabismus surgery,^[19,20]^ and it is important to note that no more than 2 recti were involved in strabismus surgery. The risk of anterior segment ischemia can be reduced by a 2-step surgical approach consisting of ciliary vessel sparing by full tendon-width transpositions or partial augmented vertical rectus transpositions.^[4,12,14,21]^Anterior segment ischemia was not present in any of our cases.

We caution against an indiscriminate use of strabismus surgery after damage to extraocular muscles from ESS as eye alignment may change during the period of weeks or months after ESS. In our series, strabismus surgery was not preformed in 10 patients, 3 of whom with a medial rectus transection with deviations of 30 to 40 PD achieved orthophoria after an average of 6 months. In other studies, spontaneous improvements were also observed within a period of 3 months after slight damage to muscle, although the mechanisms of this spontaneous improvement are not clear.^[6,12,14]^ Nonetheless, this illustrates the importance of delaying strabismus surgery for a few months in patients with slight deviations or mild rectus muscle injury.

Optic nerve injury during ESS is less common than damage to extraocular muscles and occurred in 29.6% of our patients with orbital complications. Damage to the optic nerve can occur, particularly around the posterior ethmoid sinus due to the proximity of the lamina papyracea and the optic nerve and the thin layer of fat found at this site.^[13]^ Such optic nerve damage can result from direct trauma to the optic nerve during surgery, or by indirect damage due to compression by orbital hematoma.^[13]^ Optic nerve injuries are usually rare and represent an irreversible complication. Unfortunately, no proven treatment for direct or indirect damage to the optic nerve exists.^[12]^ In our review, only 1 patient was identified with indirect trauma to the optic nerve resulting from orbital hemorrhage. Normal vision was restored in this patient after emergency decompression surgery.

Orbital hemorrhage during ESS is the most common ophthalmic complication. Han et al^[22]^ reported that 18% of retrobulbar hematomas are related to ESS. The source for orbital hemorrhage from ESS can be either arterial from damage to the anterior/posterior ethmoidal arteries or venous from injury of the lamina papyracea. Arterial hemorrhage is usually abrupt and results in rapid orbital swelling, extreme pain, visual deficits, high intraocular pressure, ophthalmoplegia, diplopia, proptosis, and periorbital ecchymosis. Venous hemorrhage is more delayed and involves slight subconjunctival bleeding, mild periorbital ecchymoses, and vision loss usually does not occur. The cause of visual deficits with orbital hemorrhage results from either vasospasm of the retinal artery or direct pressure on the ophthalmic artery. Orbital hemorrhage should be recognized and managed in a timely manner to avoid permanent visual loss.^[23]^ Systemic corticosteroids and mannitol can help in reducing intraorbital pressure and orbital swelling. If conservative treatment fails or orbital hematoma is sufficiently severe to threaten the optic nerve, canthotomy/cantholysis and endoscopic orbital decompression should be considered. Most orbital hemorrhages or hematomas from ESS were slight, related to damage of the lamina papyracea and were resolved spontaneously. In this review, all but 1 patient with mild or complicated orbital hemorrhage or hematoma were resolved within 2 weeks. This patient demonstrated arterial hemorrhage and vision deficits, but her vision returned to normal after an emergency surgery involving decompression of the orbit and optic nerve. Cerebrospinal fluid leak occurs rarely and most are cured spontaneously, ^[24]^ such as the patient in our study. A persisting leak will require surgical intervention.

In clinical practice, it would be beneficial to know the predisposing risk factors for orbital complications and the recommendations for their prevention. It is well known that the proximity of ethmoid sinus to the orbits exposes the orbital contents, especially the medial rectus, to the risk of inadvertent traumas during ESS. However, it is at greatest risk when the lamina papyracea is extremely thin and incomplete, there are anatomical variants in the sinuses, or in revision cases or cases with previous fractures. The risk of inadvertent orbital damage can be minimized by full preoperative CT review to detect preexisting anatomical variants and the extent of the sinus lesion. ^[8]^ Surgeons can reduce the incidence of orbital complications by early location of the lamina papyracea (a key landmark) and checking the position and direction of the endoscope frequently during ESS. In addition, powered instrumentation (such as its greater suction effect, its rapidly rotating sharp blades) in the sinuses also contributes to its potential for greater orbital injury. Surgeons using powered instrumentation must perform surgery with extra caution and should not point the instruments directly at the medial orbital wall.^[4]^ It is important to keep the eyes uncovered during ESS and to monitor possible orbital complications such as swelling of the eyelids, poorly reactive pupil, rapid onset of proptosis, and loss of vision. ^[23]^ This is an indication for immediate cessation of ESS.

This study has several limitations. First, it was a retrospective study, which was subject to measurement and interpretation errors. Second, it was difficult to accurately compare the effect of different surgical methods when dealing with patients who showed various ophthalmic complications following ESS. Finally, our hospital mainly enrolled patients from south China, which could introduce some geographical bias in our series.

In conclusion, ophthalmic complications during ESS include orbital wall defects, orbital hemorrhage, optic nerve damage, and extraocular muscle injury. CT evaluation should be performed in 3 planes to achieve a thorough evaluation of the injury. Medial wall defects were often small and posterior, thereby requiring no need for repair. In most of our cases, orbital hemorrhage was mild and resolved spontaneously. Optic nerve injury was usually an irreversible complication and there is no treatment for blindness after direct nerve transection. Timing and type of strabismus surgery depends on the severity and number of muscles involved as well as the type of injury. This surgery is less effective in cases of restriction factor adhesion and/or entrapment as compared to that of patients with other types of strabismus.

## Acknowledgements

The authors thank Science and Technology Program of Guangdong Province, China (Grant Numbers: 2013B021800128 and 2013B020400003) and by Science and Technology Program of Guangzhou, China (Grant Number: 15570001) for the support.
